# A Randomized, Placebo-Controlled, Double-Blind Crossover Study to Assess a Unique Phytosterol Ester Formulation in Lowering LDL Cholesterol Utilizing a Novel Virtual Tracking Tool

**DOI:** 10.3390/nu11092108

**Published:** 2019-09-05

**Authors:** Ashley Reaver, Susan Hewlings, Kenneth Westerman, Gil Blander, Thorsten Schmeller, Marianne Heer, Dietrich Rein

**Affiliations:** 1 Segterra (Inside Tracker), Cambridge, MA 02142, USA; 2 Nutrasource, Guelph, ON N1G0B4, Canada; 3 Department of Nutrition, Central Michigan University, Mount Pleasant, MI 48859, USA; 4 BASF SE, Nutrition and Health, Human Nutrition, 68623 Lampertheim, Germany

**Keywords:** phytosterol, dietary supplement, LDLc, cardiovascular disease risk, lifestyle intervention, site independent nutrition study

## Abstract

Elevated blood concentration of low-density lipoprotein cholesterol (LDLc) is a primary risk factor for developing cardiovascular disease. Lifestyle interventions including an increase in dietary phytosterols as well as medications have proven effective in lowering LDLc. The primary objective of this randomized, placebo controlled, double blind, crossover study was to determine the impact of a new phytosterol emulsion for dietary supplements (1.5 g/day phytosterol equivalents) on LDLc concentrations. Thirty-two healthy adults were randomly assigned to receive placebo or treatment followed by a washout period, followed by placebo or treatment, each phase lasting one month. Secondary endpoints related to cardiovascular health were also assessed. Study management, including screening, recruitment, monitoring, compliance, and data collection, were done remotely (a siteless clinical trial) utilizing a novel virtual tool. Phytosterol supplementation significantly lowered LDLc concentrations by 10.2% (16.17 mg/dL or 0.419 mmol/L, *p* = 0.008 by paired *t*-test, *p* = 0.014 by Wilcoxon signed rank testing). No secondary biomarkers were found to change significantly. Supplementation with phytosterols in a new dietary supplement formulation efficiently and safely decreases LDLc within one month in a free-living setting.

## 1. Introduction

Cardiovascular disease (CVD) is the leading cause of death among chronic lifestyle-related diseases worldwide. Elevated levels of total cholesterol and low-density lipoprotein cholesterol (LDLc) are primary risk factors for developing CVD [1]. Furthermore, extensive evidence suggests that lower levels of total and LDLc are associated with decreased CVD related mortality [2]. In consideration of this research, the European Society of Cardiology (ESC) and the European Atherosclerosis Society (EAS) in their joint ESC/EAS Guidelines for the Management of Dyslipidemia suggest a lower is better approach to LDLc, specifying that the greater the LDLc reduction, the greater the cardiovascular risk reduction [3]. Moreover, the benefits related to LDLc reduction are not specific for statin therapy, and no level of LDLc below which benefit ceases or harm occurs has been defined [3]. Similarly, the 2013 guidelines of the American College of Cardiology and the American Heart Association (ACC-AHA) for the treatment of cholesterol abandoned LDLc targets and advocated “the lower the better” strategy [4].

Early detection of hypercholesterolemia (HC) facilitates the implementation of effective treatment plans to lower LDLc concentration and decrease CVD risk. One routinely recommended treatment for high circulating LDLc concentrations is Therapeutic Lifestyle Changes (TLC) which involves adherence to a cholesterol-lowering diet, physical exercise, and weight management. The combination of decreasing saturated fat and cholesterol intake, increasing dietary fiber and losing weight can potentially reduce LDLc by as much as 25–35% [3,5]. Treatment with cholesterol-lowering drugs is recommended for individuals with LDLc concentrations above guidelines [2,4]. Prior to drug treatment, most recommendations identify TLC as the primary support for achieving healthy blood cholesterol concentrations and indicate that it should be the first line of defense. In reality, drug therapy is the recognized standard of care for HC [2,4]. However, consumers often seek solutions to delay the need for drug therapy. While there are several dietary options available, many are backed with questionable scientific rationale and can be confusing to the consumer.

Phytosterols may provide an option for the consumer. They are a commonly researched ingredient and are an additional recommendation to the TLC diet [2,4]. Phytosterols, also termed plant sterols, are plant-derived compounds similar in function and structure to human cholesterol [5]. In the past, phytosterols provided a larger part of the human diet, with as much as 1 g daily, as opposed to the typical modern Western diet, in which the average intake of phytosterols is about 300 mg [6,7]. Recently, there has been increasing evidence that plant-based diets can significantly lower LDLc concentrations and reduce plaque buildup in the arteries, thereby lowering the risk of CVD [8]. Consumption of phytosterols, at a level of at least 0.8 g daily, can lower blood LDLc concentrations [9]. The efficacy of phytosterols in reducing blood cholesterol levels depends upon their ability to displace dietary cholesterol in mixed micelles in the small intestine, thus hindering cholesterol absorption [6,10]. In humans, consumption of 1.5–1.8 g/day of phytosterols has been shown to reduce the absorption of cholesterol by as much as 36% [11,12].

A meta-analysis of 124 randomized controlled trials in humans, investigated the combined and separate LDLc-lowering effects of plant sterols, or phytosterols, and stanols when classified into different dose ranges [13]. LDLc-lowering effect of plant sterols and plant stanols continues to increase up to intakes of approximately 3 g/day to an average effect of 12% [13,14], and doses higher than 3 g/day of appear to be well tolerated [15]. While this is encouraging, maintaining an appropriate dietary intake to provide the daily effective dose may prove challenging to those accustomed to a Western diet. Numerous intervention studies have indicated that as little as a 10% reduction in LDLc concentrations can decrease an individual’s risk of CVD by as much as 20% [16]. If 2 g of phytosterols and stanols lowers LDLc by 10–15% this could lead to a reduction in CVD risk of 10–30% [2,3,4]. Phytosterols have also been shown to complement LDLc-lowering medications [17]. There have also been several reports of triglyceride (TG) reduction [6,18].

The most important predictor for success of a phytosterol formulation to lower LDLc is the formation of a stable micro-emulsion or a reduction of the sterol particle to a size that allows access of the sterol to the mixed micelle. The goal of this project was to create a formulation containing at least 80% phytosterol ester, which would form fine emulsions in the stomach after being released. Our approach was to create a surfactant blend that would reduce the interfacial tension leading to formation of small droplets even when only exposed to very little mechanical energy. This can only be done by reducing interfacial tension substantially, which will reduce the mechanical energy needed to break the droplets up. It is theoretically possible to choose a single surfactant that can reduce the interfacial tension enough to enable formation of very small droplets. However, an easier approach is to mix two surfactants together to achieve the right curvature of the surfactant layers, which in turn leads to optimal interfacial tension. Normally, one chooses a hydrophobic surfactant and one hydrophilic surfactant, then mixes the two surfactants until they form the suitable curvature. Our objective was to develop a new phytosterol formulation that delivers improved preconditions for effective cholesterol lowering in vivo and to substantiate the efficacy of this formulation in a clinical trial.

In this study, we tested the effect of new formulations of phytosterol ester that form fine emulsions in the stomach using a novel remote clinical study management tool. All aspects of the study were completed remotely utilizing InsideTracker, a web-based participant management platform, and Nutrasource, a contract research organization to manage the tracker application. The novel study design enabled the recruitment of a more diverse study population than traditional methods, and it allowed for daily monitoring of participant progress and compliance via the participant management system. This study is, to the authors’ knowledge, the first study to investigate dietary effects of phytosterols to be conducted without a central study location. As such, it serves as an example for future research studies utilizing remote study management.

## 2. Materials and Methods 

Our approach in developing the phytosterol formulation was split into three main steps:

a) Screen for most efficacious formulations in lab tests;
b) Test formulations in their final applications in in-vitro models;
c) Substantiate the efficacy of the best in-vitro formulation in a clinical trial.

### 2.1. Screening for Most Efficacious Formulations in Lab Tests

The objective was to develop a combination of Vegapure® 95 E sterol-ester with emulsifiers and co-emulsifiers to build a Self-Emulsifying Delivery System (SEDS). Thus, Vegapure® 95 E was used in all in vitro studies in the clinical research as the phytosterol component of the respective formulation. When given into aqueous media, the complex spontaneously forms small droplets, which enhance the bio-accessibility of phytosterol-esters for interactions with cholesterol in the mixed micelles. Furthermore, the stability of the emulsion in the stomach and the small intestine environment, compliance with regulatory requirements and a minimum phytosterol concentration of at least 80% of the formulation had to be considered. 

Using high throughput screening, we identified five hydrophobic emulsifier and five hydrophilic emulsifier blends. These were mixed until they formed the optimal curvature that would reduce the interfacial tension leading to formation of small droplets even when exposed to only very little mechanical energy. Stability in both the gastric (pH 1.6) and small intestine (pH 6.5) environments was tested. These blends were then taken to the next screening steps, which were in-vitro models.

### 2.2. In Vitro Model as First Means to Test the Effectiveness of New Formulations

CaCo-2 model: Differentiated cell cultures of human colorectal carcinoma (CaCo-2 cells) is a widespread and accepted model for in vitro studies of the human gut. We applied this model to probe intestinal transport processes, such as cholesterol assuming the following principles: Cholesterol is absorbed into intestinal cells by means of mixed micelles. Inside the cells, it is esterified, transferred to chylomicrons, and secreted to the blood stream. Phytosterols interfere with cholesterol absorption by competing for micellar solubilization. Additionally, they may have effects on the absorption site and on intracellular trafficking. In this set-up, the impact of ten different phytosterol formulations on the uptake of cholesterol into CaCo-2 cells and subsequent release into basolateral media was investigated, with a phytosterol ester formulation already on the market serving as control. 

**Figure 1:**
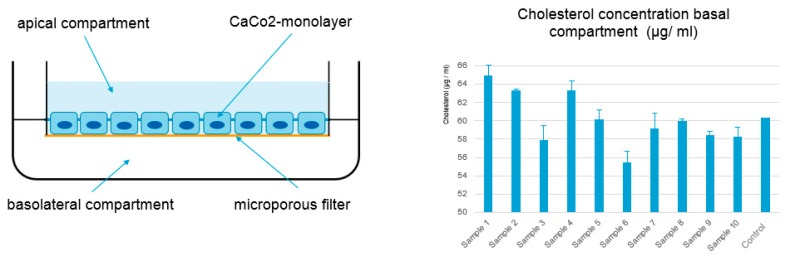
CaCo2 model: in vitro model to identify formulations with the strongest impact on cholesterol transport.

The experiments of our in vitro pretests were performed to enable decision for the best phytosterol formulation for later clinical study design. They demonstrated the inhibitory effect of different phytosterol formulations (samples 1–10) on cellular cholesterol uptake and subsequent secretion into the basolateral compartment in vitro (see Figure 1). The three formulations showing the strongest effect on reducing cholesterol concentration in the basolateral compartments were chosen for the next step of the screening program (samples 3, 6, 9). Test formulations were filled in vegan capsules (VegaGels®) and dissolution and stability trials were performed.

TIM-1 model: The TNO Gastro-Intestinal model (TIM) was chosen for the next screening step. TIM is a multi-compartmental model designed to realistically simulate conditions in the lumen of the gastrointestinal tract. Experiments in TIM are based on a computer simulation of the digestive conditions in the lumen of the gut during transit and digestion of a meal in vivo [19]. These conditions include controlled parameters such as gastric and small intestine transit, flow rates and composition of digestive fluids, pH values, and the removal of water and metabolites. The typical endpoint results obtained with TIM is the availability of a compound for absorption through the gut wall (bio accessibility). 

**Figure 2:**
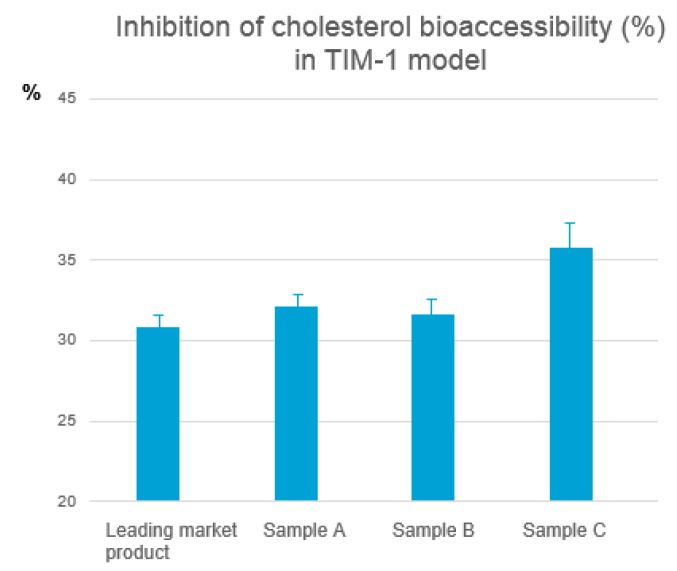
Inhibition of cholesterol bioaccessibility as measured in the TNO Gastro-Intestinal model (TIM)-1 gastro-intestinal model.

In the lumen of the intestinal compartments of TIM-1, micelles are formed due to the presence of free fatty acids (by lipase digestion of triglycerides), cholesterol, and bile salts. In the presence of phytosterols, cholesterol in the micelles can be partly replaced. Based on the data obtained from analysis of cholesterol in the micelles, the effect of different phytosterol formulations on the bio-accessibility of cholesterol can be determined. The best performing Vegapure® formulation composed of 80% phytosterol esters plus enzymatically modified sunflower lecithin, ascorbyl palmitate, and sunflower oil showed a 35.8% reduction of cholesterol bioaccessibility, (compared to 30.8% reduction seen with a leading market product, see Figure 2). The best performing micro-droplet forming phytosterol complex was subsequently tested in a clinical trial.

### 2.3. Clinical Trial Design

The primary objective was to determine the impact of one-month phytosterol supplementation on LDLc concentrations compared to placebo. The secondary endpoints were exploratory in nature and included baseline measures of HDLc, total cholesterol, triglycerides, fasting glucose, hsCRP, ALT, AST, GGT, Vitamin D, folate, vitamin B12, calcium, and magnesium for general health monitoring within InsideTracker. In addition, compliance with supplementation protocol and with lifestyle changes utilizing a novel virtual tool was a secondary aspect of the study. 

**Figure 3:**
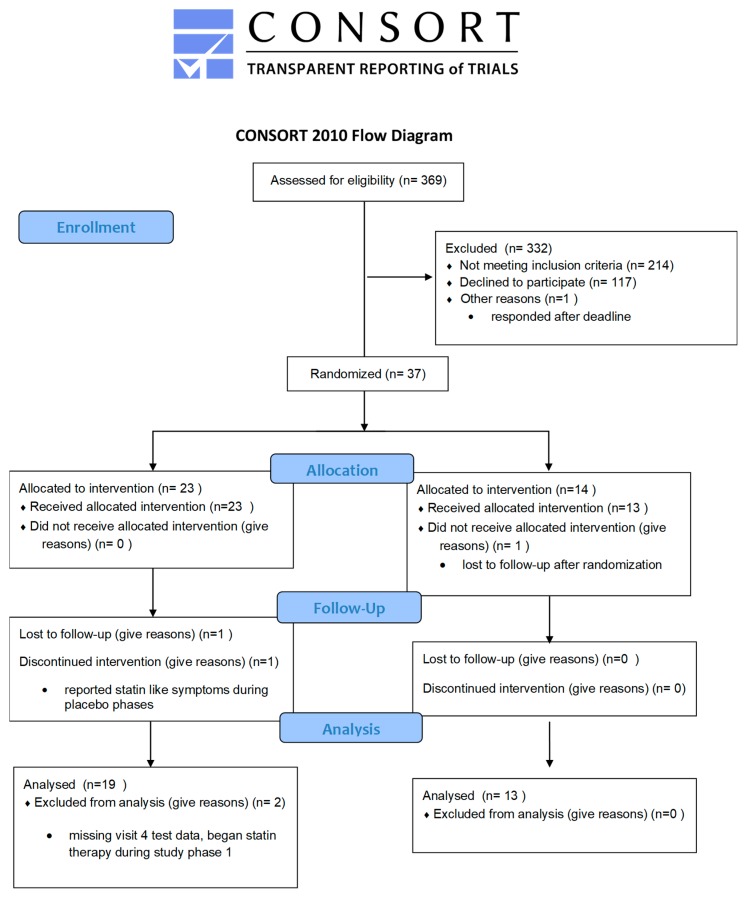
Study design flow chart of this randomized, placebo-controlled, double-blind, crossover study.

The study was a randomized, placebo controlled double blind crossover design whereby the subjects were randomly assigned to receive either placebo or intervention for one month followed by a one-month washout period followed by one month of either placebo or intervention. See Figure 3 for study design flow chart.

Study products: The phytosterol emulsion supplement (Emulsorb) consisted of Vegapure® 95E phytosterol-ester plus emulsifier in a vegan capsule (described in Section 2.1). Each phytosterol capsule had a total weight of approximately 1.6 g and was filled with 1.05 g emulsion composed of 80% phytosterol esters, enzymatically modified sunflower lecithin, ascorbyl palmitate, and sunflower oil. Each capsule contributed 0.5 g free sterol equivalent to the daily dose of 1.5 g/day phytosterol equivalent (in the form of three capsules daily) or placebo (three capsules daily). The placebo consisted of identical vegan capsules filled with approximately 1.05 g sunflower oil.

Study Population: The screening, recruitment, monitoring, compliance, lifestyle intervention, and data collection were completed remotely utilizing InsideTracker, a study participant management tool for siteless clinical trials. InsideTracker is marketed by Segterra, Cambridge, MA, USA. The platform provides blood biomarker testing, analysis, and recommendations for improving out-of-range serum biomarkers, based on a database of over 1500 lifestyle-focused recommendations curated from peer-reviewed, scientific publications [20]. Both study groups received comparable personally adjusted lifestyle recommendations during both study periods of the cross over clinical study design.

Participants were recruited from InsideTracker’s web and social network followers. Participants were assessed during the Screening phase to be a match for all inclusion criteria and absence of all exclusion criteria. Subjects were included if they were between 30–65 years of age, non-smokers, with a BMI of 20–35 kg/m^2^, had LDLc > 130 mg/dL (3.4 mmol/L), HDLc < 40 mg/dL (1.0 mmol/L) for men and < 50 (1.3 mmol/L) for women, and willingness to maintain regular activity level throughout the study. Exclusion criteria were uncontrolled high blood pressure, use of antihypertensive, anti-inflammatory, cardiovascular, or diabetic medication for at least three months prior to enrollment. Participants were enrolled through the InsideTracker platform. The InsideTracker platform interacted with all study participants in order to gather data on physical activity, diet, and lifestyle as well as provide triggers and reminders for taking the supplement and blood sampling. Informed consent was obtained from each participant prior to enrolment.

This study was conducted according to the protocol and international standards of Good Clinical Practice (International Conference on Harmonization guideline), applicable government regulations, and institutional research policies and procedures. Subjects had the right to withdraw from the clinical study at any time without explanation. The protocol was reviewed and approved by Quarum Review an Institutional Review Board, approval number QR#:32675/1.

Screening lipid panels were conducted as part of visit 1. Visit 2 included subjects who passed the screening process, signed an informed consent and were enrolled in the study. Comprehensive blood panels were taken at this time and at Visits 3–5. All collected blood samples (visits 1–5) were collected and analyzed after each collection by nationwide Clinical Laboratory Improvement Amendments (CLIA)–approved, third-party clinical labs. Participants had their samples collected in the same manner throughout the duration of the study. They were instructed to fast for 12 hours prior to the blood draw, except for water consumption. Results from the blood analysis were then uploaded to the platform via electronic integration between InsideTracker and the CLIA-approved lab. These include fasting glucose, hsCRP, ALT, AST, GGT, vitamin D, folate, vitamin B12, calcium, magnesium, ferritin, Complete Blood Count (CBC), albumin, testosterone, sex hormone-binding globulin, free testosterone (males), DHEAs (females), sodium, creatine kinase, cortisol, and potassium.

Each participant received specific lifestyle recommendations after Visit 2 These recommendations were intended to simulate lifestyle counseling that is traditionally included in clinical trials. The recommendations were generated by a rule based algorithmic expert system that used biomarker levels, demographic information, dietary restrictions, physical activity, dietary supplement regimen, and lifestyle parameters as inputs. Participants received the recommendations as short text descriptions that included the action with instruction on frequency, amount, and execution, where applicable. Exercise recommendations were provided with frequency and intensity instructions, food recommendations were provided with frequency, serving size, and recipes, and lifestyle recommendations were provided with detailed implementation descriptions specific to the recommended action.

**Figure 4:**
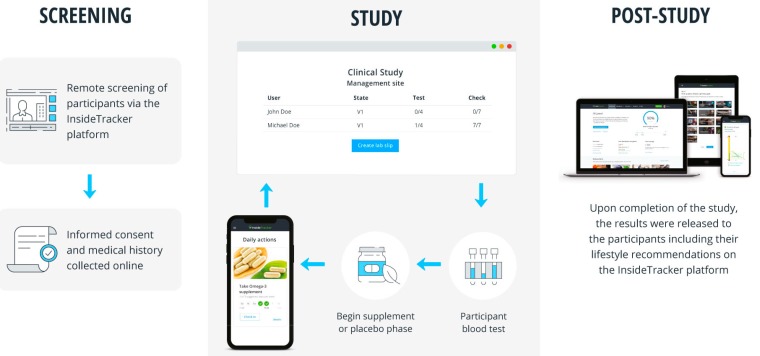
InsideTracker was used to recruit, screen, and track participants as well as to improve compliance.

Participants were instructed to consume the study product or placebo immediately prior to their two largest meal periods each day during the three distinct study phases separated by a comprehensive blood test. Compliance was assessed using a novel virtual tracking tool, InsideTracker, as well as the more traditional practice of counting any unused product (see Figure 4). Participants were asked to check-in using the virtual tool during each day of the clinical trial. InsideTracker was used as a participant portal allowing them to view their initial blood results from screening and Visit 1. As described above, the platform generated personalized nutrition and lifestyle recommendations based on visit 1 blood biomarker results to improve the out-of-range biomarkers. The use of the platform mimicked the lifestyle intervention component that often accompanies supplement clinical trials. As part of the platform, participants were required to enroll in daily check-ins and reminders. Participants set smartphone text reminders twice daily to prompt them to take their supplement or placebo before meals. At the end of each day, participants received an alert to check-in for the day. During the check-in, participants indicated whether or not they complied with instructions taking their two supplements. Participants also mailed in remaining capsules after each study phase. These capsules were counted and recorded in the data management system and utilized to measure compliance.

Confidentiality and blinding were maintained by numerical assignment. Upon enrollment, users were provided a user ID that was used as their identifier throughout the study. The InsideTracker platform automatically assigned a user ID upon enrollment in the screening process. Participants maintained this user ID throughout the study process in order to remain anonymous to the principal investigator and to maintain confidentiality. The study site kept a separate enrollment log, which matched the identifying user ID with the subject’s name and addresses. This list was confidential to the principal investigator and an operations assistant that was responsible for mailing the study materials to the subjects and coordinating with a mobile phlebotomist, if necessary. 

User IDs were used to coordinate the distribution of study materials. Study materials were labeled 0–100 by a randomization schema implemented by the study sponsor. Participant user IDs and study material IDs were maintained by the principal investigator. The sponsor confidentially secured the randomization schema until the close of the study.

### 2.4. Statistical Analysis

Changes in LDLc values during each treatment period (pre-post change during period 1 and pre-post change during period 2) were used as the main outcome of interest in an intention-to-treat analysis. Changes during each period were calculated and used in subsequent analyses. A paired *t*-test was used to test the main hypothesis of differential supplement effect versus placebo. Prior to use of the *t*-test, period and carryover effects were assessed by comparing differences across sequences and mean differences across sequences, respectively, with no statistically significant associations found. A nonparametric Wilcoxon signed rank test was also run. Secondary analyses (paired *t* -tests performed for all other biomarkers measured) were adjusted for multiple hypotheses using Holm’s method.

## 3. Results

**Figure 5:**
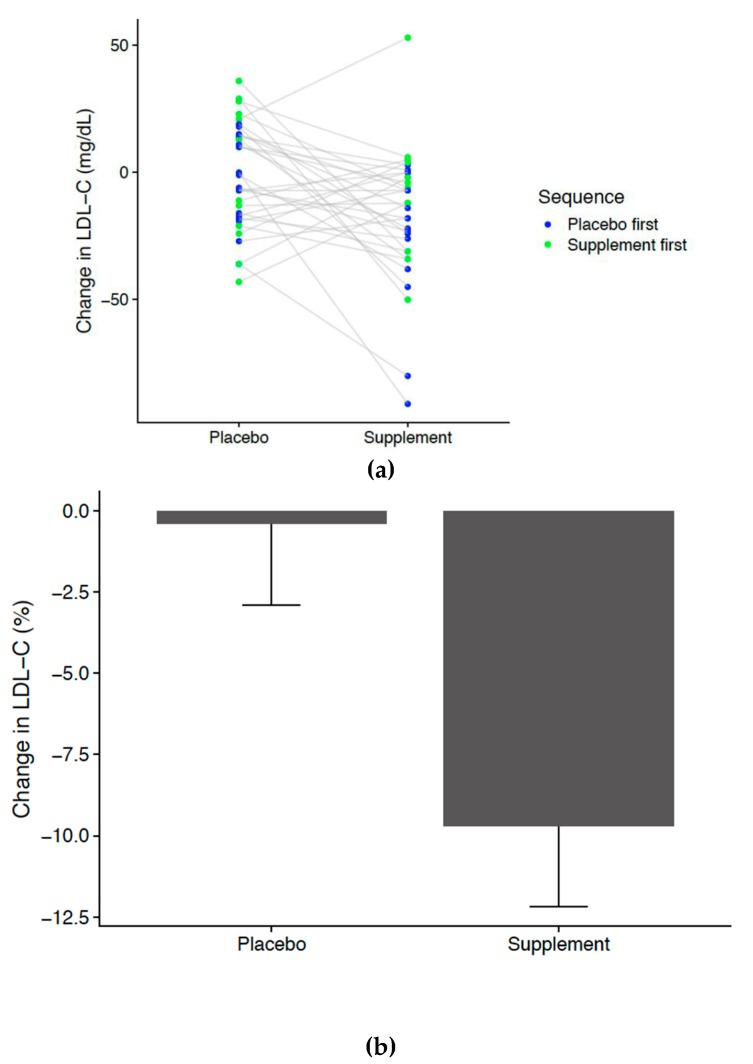
Difference in LDL concentration in placebo compared to intervention. (**a**) Change in LDLc in placebo and supplement arms. Gray bars connect changes observed in the same individual. (**b**) Mean percentage changes in LDLc in placebo and supplement arms. Error bars represent standard errors for percentage changes.

Thirty-seven non-smoking adults began the study (10 females and 27 males), 32 participants (nine females and 23 males) were included in the analysis, 13 participants received the placebo for phase 1, and 19 received the intervention for phase 1 (Figure 3). Supplements were distributed blindly and randomly by the investigators from premade kits. Since all supplement kits were not distributed, the number of participants in each group is uneven. The participants ranged in age from 30–65 with an average BMI of 25.8 kg/m^2^ (range 20–40). The main intention-to-treat analysis using a paired *t*-test for response change during supplement vs. placebo showed a significant change (difference in LDLc changes of 16.2 mg/dL, *p* = 0.008) a reduction of 10.2% (Figure 5) after the one-month supplement phase of the trial. Nonparametric supplementary analyses of this change demonstrated qualitatively similar results: a nonparametric Wilcoxon signed rank test for the same difference showed *p* = 0.014). The mean pre-supplement phase LDLc levels were not different between the placebo-first and the supplement–first groups (*p* = 0.19), and the mean post-supplement phase LDLc changes were also not different (*p* = 0.13). No secondary biomarkers including fasting glucose, hsCRP, ALT, AST, GGT, vitamin D, folate, vitamin B12, calcium, or magnesium was found to be significant after multiple hypothesis correction (Supplementary Table S1).

### 3.1. Compliance

The study had a low rate of attrition. Only three of the 37 subjects did not finish the study. One was lost to follow-up immediately after enrollment, another was lost to follow-up after phase 1, and the third withdrew from the study due to perceived adverse events (discussed in Section 3.2). Of those that completed the study, only nine subjects completed the study with compliance of greater than 80%. Failure to comply was measured as failure to mail in remaining supplements after all three study phases (subjects were provided post-paid packaging for return) or greater than 20% of supplements returned for any one study phase. The majority of non-compliant subjects failed to mail in the remaining supplements after all three study phases. Compliance was also monitored via the InsideTracker application. Participants checked in twice daily for completing the AM and PM supplement regimen. This measure was not included in the determination of compliance because it could not be verified as correct. Remaining pill count was the only metric included in the determination of compliance.

### 3.2. Safety

Overall, the investigational product was well tolerated by subjects. Four adverse events were reported by participants. Three resolved on their own (funky after taste/dry mouth, photophobia, constipation). The fourth participant reported statin-like symptoms such as cognitive and erectile dysfunction while on placebo. The participant withdrew from the study before beginning the investigational supplement.

## 4. Discussion

The first goal of this project was to create a formulation containing at least 80% phytosterol ester, which would form fine emulsions in the stomach after being released. Our approach was to create a surfactant blend that reduces the interfacial tension leading to formation of small droplets even when only exposed to very little mechanical energy. The phytosterol formulation developed for this study delivers optimal conditions for effective cholesterol lowering in vivo and substantiates the efficacy of this formulation in a clinical trial. The reduction in LDLc seen in our clinical trial provides evidence for the efficacy of this formulation.

The 10.2% reduction in LDLc achieved with the new Vegapure® 95E phytosterol emulsion in this study is significant and clinically meaningful when compared to previous investigations testing the dietary supplement format [13,14,21]. Limited data is available on the LDLc-reducing effects of dietary supplements containing plant sterols and stanols. Results show a large variation of the LDLc lowering effect [13,14]. Considering the limited comparability among studies, it is still remarkable that the LDLc-lowering effect in the current study was about 25% larger than the average of previous studies extrapolated to a 1.5 g of plant sterols or stanols per day as dietary supplement intake, indicating improved efficacy of this formulation.

In the meta-analysis by Katan et al. 2003 [15] a 1.5–1.9 g/day plant sterol or stanol intake resulted in an average 8.5% decrease in LDLc. Whereas the meta-analysis of Musa-Veloso et al. 2011 [22] showed a 7.7% decrease with 1.8 g sterol or stanol per day and Ras et al. 2014 [13] observed a 7.6% LDLc reduction in their meta-analysis. The LDLc reduction observed in our study could be in the order of a 10-20% reduction in risk of CVD [15]. To further elucidate the benefits of such LDLc lowering, a meta-analysis reported that a 1 mmol/L (39 mg/dL) reduction in LDLc provides a 27% decrease in the relative risk of experiencing any CHD-related event and a 28% decrease in the relative risk of a CHD attributed death [23]. A similar meta-analysis confirmed a dose-response relationship between food sources of phytosterols and LDLc lowering [9].

Furthermore, a recent review concluded that evidence from long term studies supports the safety and efficacy of plant sterols thus supporting their use in cholesterol lowering either alone or in combination with pharmacotherapy [24]. The combination use is supported by the results of a meta-analysis that, after reviewing 15 randomized controlled trials of 500 participants, concluded that sterol- or stanol-enriched diets lower total cholesterol and LDLc concentrations in patients treated with statins beyond that achieved by statins alone [25]. A meta-analysis of eight randomized controlled trials evaluated the use of plant sterols/stanols in combination with statin therapy in individuals with hypercholesterolemia and observed significant decreases in total cholesterol and LDL cholesterol but not HDL cholesterol or triglycerides [26].

Despite the pronounced and significant LDLc reduction observed in this study (16.17 mg/dL or 0.419 mmol/L, *p* = 0.008 by paired *t*-test), Figure 5a shows that not all individuals appeared to benefit from phytosterol supplementation. A small percentage of the population with high basal cholesterol synthesis is less responsive to phytosterol treatment than are subjects with low basal cholesterol synthesis [27]. This heterogeneity in response may in part be explained by individuals compensating reduced cholesterol absorption with increased endogenous synthesis [28]. Thus, future dietary phytosterol supplementation may be even more effective in reducing LDLc once metabolic and genetic variation can be accounted for in individualized health plans.

The reduced risk from phytosterol intake may translate into significant economic savings. The connection between lower LDLc concentrations and the decrease of CVD events would ultimately translate into a decrease in overall CVD event risk and therefore CVD-attributed costs at an expected rate of about $140 million per year [29]. In the United States, use of phytosterols in adults would potentially lead to a health care costs saving of $4.2 billion on average per year—a cumulative total savings of $34.0 billion from 2013 to 2020 [30].

In addition to a clinically significant reduction in LDL cholesterol, this study demonstrates the utility of a study management tool, to conduct screening, recruitment, monitoring, compliance, and data collection. This was all done remotely utilizing a novel virtual tool, InsideTracker. The novel study design enabled the recruitment of a more diverse study population than traditional methods because location was not a limiting factor in recruitment. Furthermore, the population of InsideTracker users from which the participants were selected are generally healthy and health-conscious. It may have contributed to the low attrition rate of study participants, as only three subjects did not finish the study. Personalization of text messages for supplement reminders may have kept participants engaged longer than traditional studies. Additionally, the platform allowed for daily monitoring of participant progress and compliance via the participant management system with very low human capital cost. The principal investigator contacted subjects if they did not check into the platform for several days, which again may have aided in the low rate of attrition. Furthermore, participants were able to interact with the InsideTracker platform, which allowed them to interact with the lifestyle recommendations provided after their first blood analysis. Participants were permitted to view specific foods and exercise information pertinent to their blood biomarkers. Unlike traditional lifestyle counseling conducted in supplement studies, access to a web-based platform that displayed interactive recommendations may have captured and held participants’ interest for the duration of the study. 

This study is the first phytosterol study to the authors’ knowledge to be conducted without a central study location. As such, it serves as an example for future research studies utilizing remote study management. A potential benefit to utilizing such a virtual tool is the interaction and reminders sent to subjects. Subject attrition rate is a common challenge in experimental studies, especially longer studies. It has been predicted that a 20% loss to follow up can threaten validity and increase risk of bias [31]. Therefore, several strategies have been tested in the literature to address the issue. These include pre-calling, email reminders, personalizing communication, and incentivizing and increasing subject interaction [32,33]. Compliance is an important aspect of experimental trials as such. This is challenging when one considers that adherence to chronic disease medication has been reported to be just 50% [34]. Strategies to improve treatment compliance include facilitating communication, patient education, distribution of reminders, and offering support, all of which the virtual tool can address [35]. Our study faced typical compliance issues in that of those that completed the study, only nine subjects completed the study with full compliance. Failure to comply was measured as failure to mail in remaining supplements after all three study phases or greater than 20% of supplements returned for any one study phase. The majority of non-compliant subjects failed to mail in the remaining supplements after all three study phases despite receiving pre-paid envelopes for all returns. Though, to our knowledge, no studies have tested the full scope of features offered by this virtual tool, it does provide many of the suggested techniques for decreasing attrition rates. Future studies should consider utilizing and thoroughly investigating a comprehensive virtual tool.

### Limitations

One limitation of the study is the low number subjects, although this is not uncommon in dietary supplement trials. Future studies should consider including a larger subject number. That being considered, we do believe the cross-over design of this study to be a strength. Though the study design allowed for enhanced compliance monitoring and thus transparency, we did experience relatively low compliance. Strategies to enhance this should perhaps be considered for future studies. We speculate that the distance may have had an impact on our compliance metrics of mailing in remaining supplements. Had participants been required to visit a study center for each blood draw, collection of the supplements may have been more likely. Another limitation is that the study does not control for diet. Although we did not control for dietary changes, it was intentional to mimic real-life scenarios. While carryover effects were tested for and confirmed to be mild in this sample, it is possible that the results could be affected by serum phytosterol concentrations remaining somewhat higher than typical after the washout period in participants receiving the supplement-first sequence. Finally, the study subjects recruited from InsideTracker database may be biased toward their health interest, but this reflects the real-life consumer base for phytosterol supplements.

## 5. Conclusions

The new phytosterol emulsion in the form of capsules is effective in lowering the cardiovascular risk factor LDLc among mildly hypercholesterolemic subjects in a remotely monitored setting of free-living subjects. The significantly improved LDLc (−10.2%) was reached after one month of administration. This is about 25% more than expected from the same amount of phytosterol dietary supplement capsule or tablet. In addition, this study provides support for the use of a novel virtual tool to enhance study management.

## 6. Patents

The formulation is covered by the US patent 10,188,133 B2 with the title “Gel capsule containing sterol and solubilizing agent,” which also describes the in vitro results.

## Supplementary Materials

The following are available online at https://www.mdpi.com/2072-6643/11/9/2108/s1, Table S1: Supplement vs. placebo.
