# Supplementary material for: A Randomized, Placebo-Controlled, Double-Blind Crossover Study to Assess a Unique Phytosterol Ester Formulation in Lowering LDL Cholesterol Utilizing a Novel Virtual Tracking Tool
Source: Nutrients. 2019 Sep 5;11(9):2108. doi: 10.3390/nu11092108 (PMC6769481; doi:10.3390/nu11092108)
Supplement: Supplementary file 1 [file nutrients-11-02108-s001.pdf]

Changes in other biomarkers

Results below reflect paired t-test results for each secondary biomarker (multiple testing correction using Holm's method).

Table 1: Supplement vs. placebo

Biomarker	Mean abs. change	Mean % change	T-test p-value	Corrected p-value
Mg	-0.08	-4%	0.0027	0.1280
LDL	-16.16	-10%	0.0076	0.3566
Chol	-21.09	-9%	0.0088	0.4054
Fol	-2.47	-15%	0.0456	1.0000
HDL	-3.44	-6%	0.1086	1.0000
Lymphocytes	87.81	5%	0.1435	1.0000
TIBC	-9.59	-3%	0.1480	1.0000
AST	2.66	12%	0.1668	1.0000
Platelets	9.09	4%	0.1741	1.0000
Monocytes %	-0.57	-7%	0.1751	1.0000
B12	-42.25	-7%	0.1841	1.0000
CK	74.94	40%	0.2231	1.0000
ALT	2.16	10%	0.2293	1.0000
WBC	0.28	5%	0.2330	1.0000
Basophils %	-0.08	-10%	0.2393	1.0000
Ferritin	7.59	6%	0.2773	1.0000
Neutrophils	192.31	7%	0.3627	1.0000
Fe	-9.44	-8%	0.3651	1.0000
Sodium	0.44	0%	0.3704	1.0000
Cortisol	-0.98	-7%	0.4271	1.0000
Glucose	1.25	1%	0.4319	1.0000
MCH	-0.08	0%	0.4734	1.0000
DHEAS	-10.25	-9%	0.4829	1.0000
TS	-2.00	-6%	0.4887	1.0000
Tes	-11.34	-3%	0.4895	1.0000
GGT	0.78	4%	0.4902	1.0000
RBC-Mg	-0.07	-1%	0.4912	1.0000
RDW	0.07	1%	0.5560	1.0000
Free testosterone	-0.28	-4%	0.5622	1.0000
Albumin	-0.03	-1%	0.5936	1.0000
Monocytes	-9.12	-2%	0.6690	1.0000
hs-CRP	0.35	23%	0.7134	1.0000
Basophils	-1.00	-2%	0.7440	1.0000
Eosinophils	4.84	4%	0.7497	1.0000
Neutrophils %	0.53	1%	0.7676	1.0000
Vitamin D	0.45	1%	0.7802	1.0000
MCHC	-0.05	0%	0.7902	1.0000
TG	2.00	2%	0.8187	1.0000
Calcium	-0.02	0%	0.8269	1.0000
Eosinophils %	-0.05	-2%	0.8446	1.0000
RBC	0.01	0%	0.8608	1.0000
Hemoglobin	-0.03	0%	0.8709	1.0000
MCV	-0.08	0%	0.8728	1.0000
Hematocrit	-0.07	0%	0.8808	1.0000
Lymphocytes %	0.14	0%	0.9233	1.0000
